# Naturally occurring drug resistance associated variants to hepatitis C virus direct-acting antiviral agents in treatment-naive HCV genotype 1b-infected patients in China

**DOI:** 10.1097/MD.0000000000006830

**Published:** 2017-05-12

**Authors:** Zhanyi Li, Ying Zhang, Ying Liu, Xiaoqiong Shao, QiuMin Luo, Qingxian Cai, Zhixin Zhao

**Affiliations:** a Department of Infectious Diseases, Third Affiliated Hospital of Sun Yat-sen University; b Guangdong Provincial Key Laboratory of Liver Disease, Guangzhou, Guangdong, China.

**Keywords:** direct-acting antiviral agents (DAAs), hepatitis C virus genotype 1b, NS3/4A, NS5A and NS5B genes, resistance-associated variants (RAVs)

## Abstract

The direct-acting antiviral agents (DAAs) have drastically improved the prognosis of hepatitis C virus (HCV) patients. However, the resistance-associated variants (RAVs) to DAAs may hamper treatment. There was a lack of data on the prevalence of pre-exist RAVs in Chinese HCV-infected patients. We performed nested PCR assays on 74 HCV genotype 1b-infected patients to amplify HCV viral regions of NS3, NS5A, and NS5B to investigate the prevalence of RAVs to DAAs in treatment-naive HCV genotype1b-infected patients in China. The mutations A156S, T54S, and D168Y of the NS3/4A region were found in 18.33% (11/60), 6.67% (4/60), and 1.67% (1/60) of the successfully amplified cases. Mutations Q30R, L31M, and H58P of the NS5A region were confirmed in 57.63% (34/59), 1.69%(1/59), and 86.44% (51/59) of the cases. Mutations C316N, S365A, M414L, M423I, Y448H, I482T, I482 V, V494L, P495S, and V499A of the NS5B region were detected in 100% (60/60), 3.33% (2/60), 5.88% (3/51), 1.96% (1/51), 1.96% (1/51), 5.88% (3/51), 1.96% (1/51), 3.92% (2/51), 5.88% (3/51), and 15.69% (8/51) of cases, respectively. Naturally occurring RAVs to DAAs pre-exist in treatment-naive Chinese HCV genotype 1b-infected patients and the characteristic is different from that in Europe and the United States. Clinicians should consider RAVs upon the introduction of DAA-based antiviral therapy.

## Abstract 2

Supplemental Digital Content is available in the text

## 1 Introduction

Hepatitis C virus (HCV) has infected more than 185 million people globally, among whom 350,000 die each year. One-third of those who become chronically infected are predicted to develop liver cirrhosis, hepatocellular carcinoma.^[1]^ HCV infection is an important cause of liver transplantation due to end-stage liver disease.^[2]^ In China, there are about 38 million patients with chronic hepatitis C (CHC), most of whom are infected with HCV genotype 1b.^[3]^ CHC has become a global health problem and poses a serious health burden.

Polyethylene glycol interferon (PEG-IFN) along with ribavirin (RBV) were recommended as the standard of care (SOC) for patients with CHC before 2011. However, the various genotypes and subtypes of HCV have been associated different response rates with the SOC. It was reported that patients infected with HCV genotypes 2 and 3 could achieve a sustained viral response (SVR) rate of more than 70% to 48 weeks of PEG-IFN/RBV treatment, while patients with HCV genotypes 1 could only get no more than 50%.^[4]^ However, the treatment of CHC is burdened by the adverse effects of PEG-IFN and RBV. Parts of the CHC patients have the interferon contraindications before the treatment. More effective and safe treatments are urgently required.

As development in the knowledge of molecular biology of HCV life cycle, several molecules that specifically block various viral proteins were discovered.^[5,6]^ These compounds are known as direct-acting antiviral agents (DAAs) and target various viral nonstructural proteins, including the NS3/4A protease, the NS5A protein, and the nucleosides /non-nucleoside NS5B polymerase. Some of them have already been approved for the treatment of HCV infection.^[1,7,8]^ Many studies had reported that DAA regimen exhibited potent antiviral activity with high SVR rate and insignificant side effects, even in difficult-to-treat patients including old patients, patients with a liver cirrhosis, and those who failed to previous PEG-IFNα/RBV treatment.^[9–13]^ However, the high replication rate of HCV and the low fidelity of its polymerase combined with selective pressures by the immune system and drug treatment resulted in a sequence variation in the HCV population, leading to a quasispecies and the potential selection of drug resistance-associated variants (RAVs).^[14,15]^ Recently, the mutations with varying degrees of drug resistance to DAAs^[16–21]^ had been detected, even in DAA-naive patients and caused primary drug resistance.^[22–24]^ The presence of these RAVs can limit the efficacy of DAAs and the substitutions of amino acid in the targeted proteins affect viral sensitivity to DAAs.^[15]^ The presence of RAVs in HCV can hamper the treatment of DAAs and has raised public health concerns.

DAAs are either unavailable or unaffordable in Mainland China. Most HCV-infected patients were DAAs-naive. There was a lack of data on the prevalence of pre-exist RAVs in Chinese HCV-infected patients. Because of the different HCV quasispecies, it is probable that the characteristics of RAVs to DAAs were different from precious studies. The object of this study was to investigate the prevalence of RAVs to DAAs in treatment-naive HCV genotype1b-infected patients in China.

## 2 Methods

### 2.1 Patients

Eight hundred seventeen HCV patients who were admitted into the third affiliated hospital of Sun Yat-sen hospital between 2009 and 2012 had been genotypes. Seventy-four HCV genotype1b-infected patients who were treatment naive were consecutively enrolled. The diagnosis of HCV was based on guidelines on the prevention and treatment of hepatitis C approved by American Association for the Study of Liver Disease. The patients coinfected with HBV or HIV and those had other liver diseases such as alcoholic hepatitis were excluded. All the patients are Chinese Han population. The study protocol was approved by ethics committee of the third affiliated hospital of Sun Yat-sen University and the informed consent document was obtained from each patient.

### 2.2 RNA extraction, reverse transcription, and quantification

RNA was isolated from the first RNA-positive serum sample obtained from each patient using 500 μL serum and aRNAiso Plus extraction kit (Takara, Dalian, China). The HCV RAN was quantified by detecting the light absorption value using the trace nucleic acid analyzer (Thermo, CA) at the wavelength of 260 nm. HCV RNA was eluted in 10 μL of Tris-EDTA (TE) buffer and was subsequently transcribed into cDNA using the ReverTra Ace-α-reverse transcription kit (Toyobo, Shanghai, China). This cDNA was used as the input for 2 separate PCR assays targeting the HCV core and NS5B regions.

### 2.3 Genotyping methods

Five milliliters of peripheral blood was taken from HCV patients, and RNA was extracted using Omega Viral RNA Kit (Tiangen, Beijing, China). And then, the obtained cDNA was synthesized from RNA with Toyobo ReverTra Ace-αreverse transcription kit (Tiangen, Beijing, China). HCV genotyping was performed using our developed method as previously described.^[25]^ In brief, nested PCR was utilized to amplify the conserved fragments of genes HCV core and NS5B. The obtained HCV core and *NS5B* gene were sequenced, and compared with the existed HCV sequence to identify the genotype. According to the gene sequences of HCV NS3, HCV NS5A, HCV NS5B in GenBank, we designed the specific nested PCR primers for HCV1b. The primers were listed in supplemental Table 1 to Table 4. The polymerase chain reaction (PCR) was carried out with Thermal Cycler S1000 PCR machine (Thermo, CA), and the reaction conditions were reported in our previous literature.^[25]^ Clustal X was used to perform sequence alignment.

### 2.4 Sequence alignment and analysis

The gene sequence was compared using the Clustal X program. The NS3/4A, NS5A, and NS5B mutations were analyzed and blasted with the mutations reported in the previous studies. The blast was performed according to the output peak chart produced by ABI 3730xl DNA Sequencer (ABI, Carlsbad, CA).

### 2.5 Statistical analysis

SPSS 19.0 (IBM, Armonk, NY) was employed to perform statistical analysis. The clinical characteristics are presented as percentage, means with standard deviations (SD) or median (minimum, maximum), and 2-tailed Student *t* test; nonparameters, 1-way analysis of variance (AVOVA) and the Mann–Whitney *U* test were adopted to determine the statistical difference, and *P* < .05 was considered to be significant.

## 3 Results

### 3.1 Characteristics of HCV 1b infected patients with or without mutations

In the mutation type group, the mean age was 38.52 ± 13.22 years, 63.64% of the patients were male, and the mean HCV viral load was 7.09 ± 0.71 IU/mL log_10_. In the wild-type group, the mean age was 34.29 ± 15.79 years, 62.50% of the patients were male and the mean HCV viral load was 6.75 ± 0.33 IU/mL log_10_ (Table 1). There was no significant difference among the patients with or without mutation (*P* > .05) for clinical characteristics, such as age, aspartate aminotransferase (AST), alanine aminotransferase (ALT), albumin (ALB), platelet (PLT), hemoglobin (Hb), body mass index (BMI), and HCV RNA levels. There was no significant difference among the patients with NS3/4A, NS5A, and NA5B mutation (*P* > .05) for clinical characteristics, such as age, AST, ALT, ALB, PLT, Hb, BMI, and HCV RNA levels (Table 2).

**Table 1:**
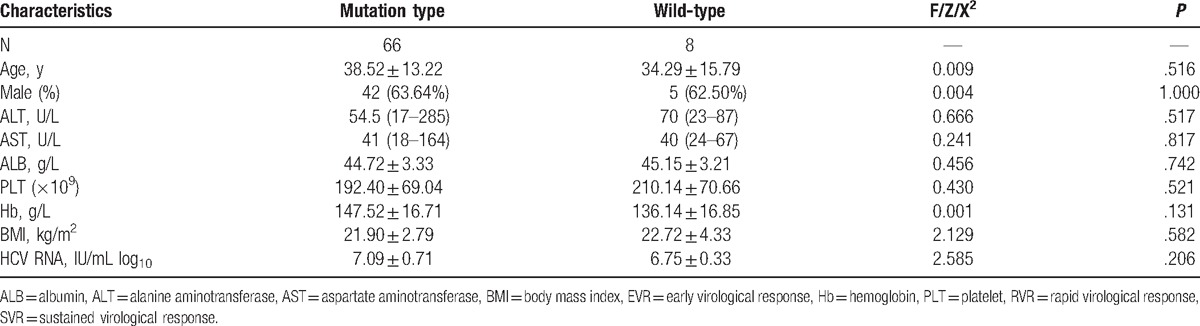
Characteristics of HCV 1b-infected patients with or without RAVs.

**Table 2:**
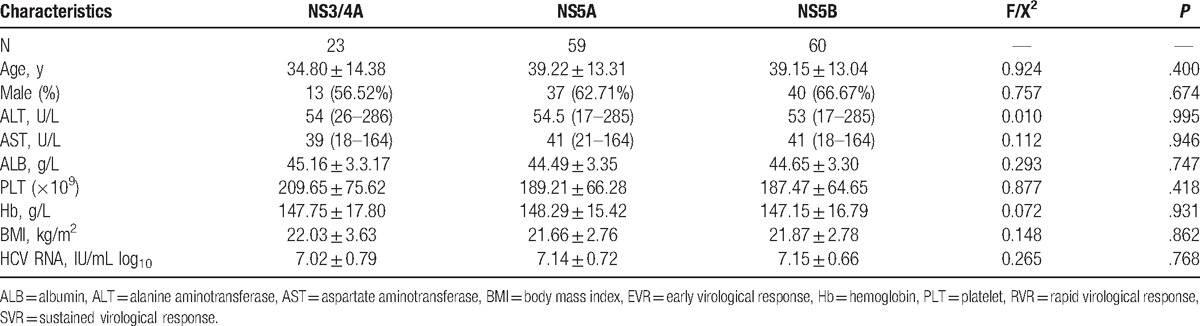
Characteristics of HCV 1b infected patients with mutations.

### 3.2 Analysis of RAVs to NS3/4A protein protease inhibitors (PIs)

The success rate of amplification of NS3 was 81.08% (60/74). The mutation rate was 38.33% (23/60). There were 11 cases (18.33%, 11/60) with the main mutation A156S associated with resistance to Asunaprevir, Boceprevir, Paritaprevir, Simeprevir, and Telaprevir. There were 4 cases (6.67%, 4/60) with the mutation T54S associated with resistance to Boceprevir and Telaprevir and 1 case (1.67%, 1/60) with mutation D168Y associated with resistance to Asunaprevir, Paritaprevir, and Simeprevir. The frequency of V170I was 16.7% (9/60) in HCV genotype 1b. Only 1 case (1.67%, 1/60) with the mutation V55R was found in HCV genotype 1b infected patient when 2 cases (3.33%, 2/60) with the mutation Q80L were found in HCV genotype 1b infected patients (Table 3).^[23,26–31]^

**Table 3:**
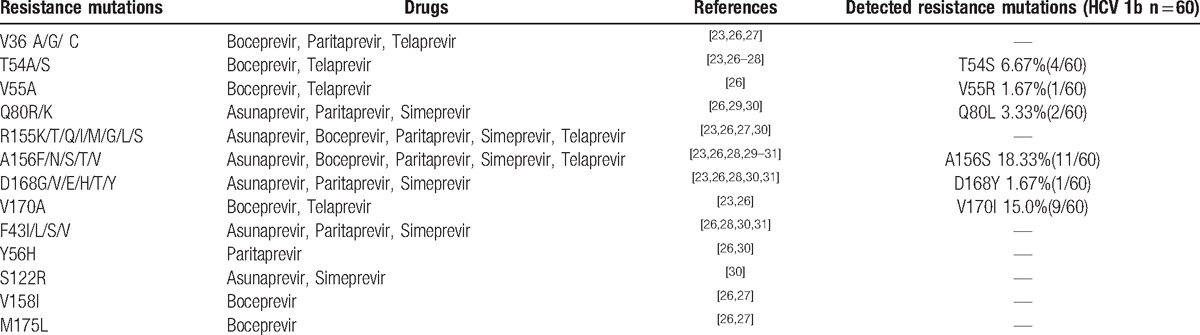
The outcomes of RAVs to HCV NS3/4A PIs.

### 3.3 Analysis of RAVs to NS5A

The success rate of amplification of NS5A was 79.7% (59/74). The mutation rate was 100% (59/59). There were 34 cases (57.6%, 34/59) with Q30R mutation associated with resistance to Daclatasvir, Ombitasvir, and Ledipasvir, while there were 51 patients (86.4%, 51/59) with H58P associated with resistance to Daclatasvir and 3 cases (5.08%, 3/59) were detected Y93H mutation associated with resistance to Daclatasvir, Ombitasvir, and Ledipasvir. Other mutation sites such as M28L, H54Q, H58T, H58S, H58R, Y93T, and Y93A that were not been proven to correlate with the drug-resistant properties in previous studies (Table 4).^[17,26,30,32–35]^

**Table 4:**
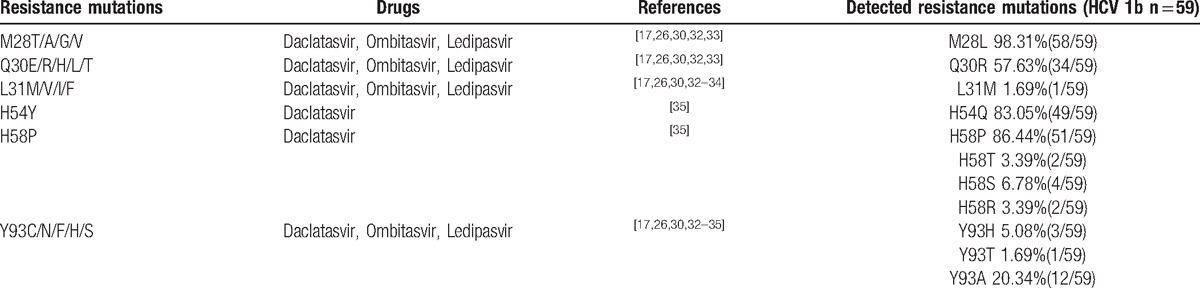
The outcome of RAVs to HCV NS5A inhibitors.

### 3.4 Analysis of RAVs to NS5B

Due to the high difficulty to amplify the fragments of NS5B, the fragments of NS5B were divided into 3 fragments, and then amplification was performed. The first fragment contained A15 and S96. The second fragment contained C223, S282, C316, V321, S365, and S368. The third fragment contained M414, L419, M423, Y448, I482, V494.

The success rate of amplification for the first fragment was 93.2% (69/74). Among the successfully amplified samples, no patients had the drug resistance mutation. For the amplification of the second fragment, the success rate was 81.08% (60/74), while the mutation rate was 100% (60/60). These mutations are associated with resistance to Dasabuvir, Tegobuvir, and HCV796. For the amplification of the third fragment, the success rate was 68.92% (51/74), while the mutation rate was 47.05% (24/51). These mutations were associated with resistance to Dasabuvir, Tegobuvir, HCV796, JTK-109, and Deleobuvir (Table 5).^[36–38]^

**Table 5:**
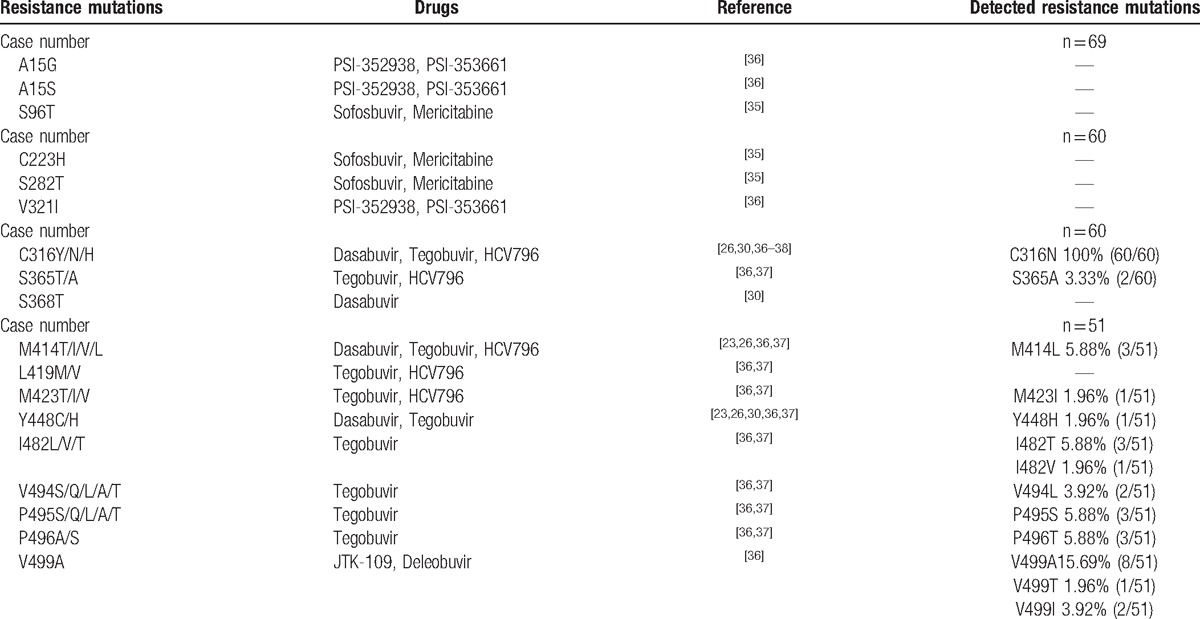
The outcome of RAVs to NS5B inhibitors.

### 3.5 Analysis of multiple RAVs

We also found that 59 patients have 2 or more than 2 RAVs that will result in highly resistant toward DAAs and resistant to multiple DAAs. There were 12 cases which had 3 to 5 RAVs, which will result in resistant toward NS3/4A inhibitor, NS5A inhibitor, and NS5B inhibitor. There were 38 cases that had 2 to 5 RAVs, which will result in resistant toward NS5A inhibitor and NS5B inhibitor. Two cases had RAVs that will result in resistant toward NS3/4A inhibitor and NS5B inhibitor. At the same time, we found that 4 cases had Q30R+ H58P that results in resistance to NS5A inhibitor: one case with C316N + V499A, one case with C316N + M423I, and one case with C316N + I482T, which is resistant to NS5B inhibitor (Table 6). There was linkage disequilibrium in our patients.

**Table 6:**
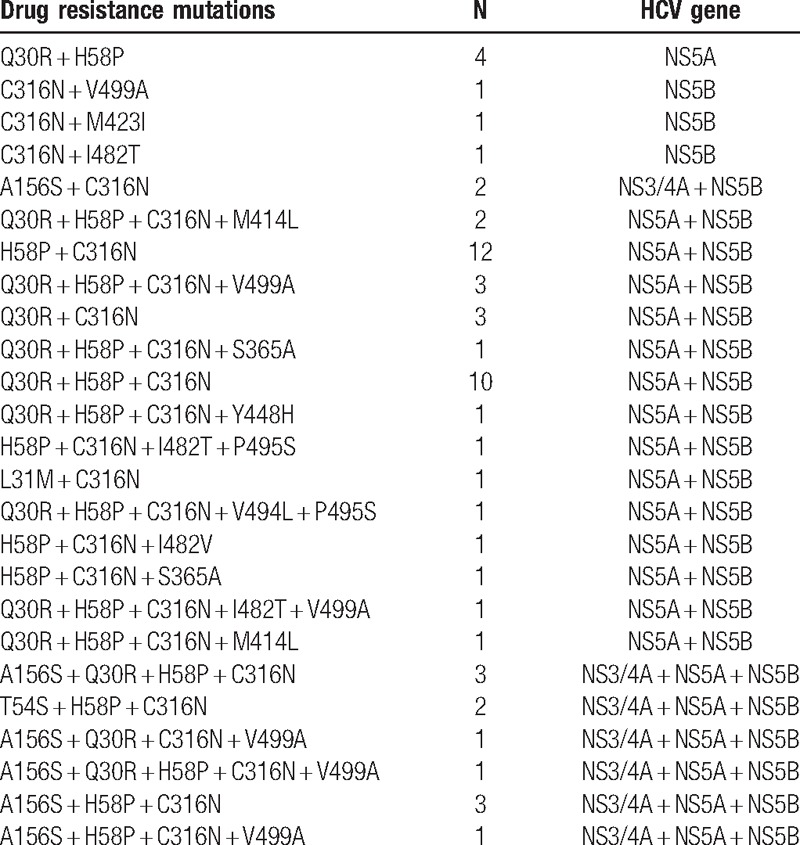
The outcome of multiple RAVs to DAAs.

## 4 Discussion

The development of DAAs represents a significant advancement in HCV antiviral therapy. Despite the antiviral potency of the majority of DAA being extraordinary, the ability of HCV to rapidly evolve in the setting of drug pressure and the presence of baseline natural polymorphisms associated with resistance to drugs must be considered as possible challenge to the success of these therapies. Clinical trials had already showed that the RAVs could lead to treatment failure and these RAVs could be found in DAAs treatment-naive HCV patients.

It had been reported that HCV NS3 was a multifunctional antiviral target exhibiting large gene polymorphisms. It had been detected that the main sites (R155 and A156) had less variation, whereas the second sites (V36, T54, Q80, D168, V170) had variation more frequently. Our study showed that 26.67% (16/60) patients infected with HCV genotype 1b had the drug resistance mutations to NS3/4A PIs before any direct antiviral treatments. The prevalence of 26.67% (16/60) for PI resistance proven patients observed in the present study was higher than the results of previous studies.^[23,24,26]^ As compared with the previous studies, main mutations such as R155, A156T, Q80K, those that may result in high drug resistance, were not detected in our study, while D168Y that may result in high drug resistance was detected in our study. The Q80K variant was associated with different levels of resistance to some approved NS3 PIs (asunaprevir, paritaprevir, and simeprevir). Patients harboring the Q80K had lower SVR rates to simeprevir than those who did not. SVR rates in simeprevir-based treatment-naïve HCV genotype 1a infected patients with and without the Q80K variant were 58% versus 84%^[23,28]^ and guidelines recommended to screen for presence of Q80K before beginning the simeprevir drug therapy and to consider alternative therapy if the Q80K are detected.^[1,7,8]^ Variations A156S and T54S were found in 11 cases and 4 cases, while some invalid variations were found, including V170I, V55R, and Q80L, which have not been proved to be drug resistant.

NS5A PIs, a new type of direct-acting antivirals, interfered HCV replication cycle mainly through directly inhibiting NS5A. Compared with the majority of NS3 RAVs, variants conferring resistance to NS5A inhibitors are generally more frequently detected as natural variants in HCV genotype 1 infected DAA-naive patients.^[39]^ As a single drug resistance mutation, the rate of natural occurrence was estimated between 0.3% and 2.8% in different studies by population sequencing.^[26,30,32,39]^ In our study, the rate of natural occurring mutations was higher. For instance, the mutation rate of Q30R was 57.63% (34/59), while H58P was 86.44% (51/59). Y93H that confers medium to high-level resistance to the 3 approved NS5A inhibitors (Daclatasvir, Ombitasvir, Ledipasvir) seems to be less frequent in Chinese HCV genotype 1b infected patients (5.08%) than the European (15.0%) and the US (9.3%).^[32]^ Four patients had dual combinations of mutations Q30R+H58P that had never been investigated and their level of resistance was unknown.

The HCV NS5B is the last nonstructural gene sequence of HCV, and is located in the end part genome of the HCV. The variation of NS5B amino acid sequence can influence DAAs antiviral capacity and resistance of genetic barrier. The RAVs C316N was detected in all of our patients who were never treated by the DAAs. Whereas main mutations such as S282T that may result in high drug resistance were not detected in our study. S282T that confers high-level resistance to sofosbuvir^[26]^ may result in virologic relapse and sofosbuvir-containing regimens treatment failure.^[9,26]^ The RAVs of non-nucleoside inhibitors of NS5B were more frequent than that of nucleoside inhibitors of NS5B. In our patients, no RAVs to NS5B nucleoside inhibitors was detected, whereas 100% patients had the RAVs of non-nucleoside inhibitors, including C316N, S365A, M414L, M423I, Y448H, I482T/V, V494L, P495S, V499A. Interestingly, HCV genotype 1b isolates harboring C316N were more frequently observed in Chinese patients (100%) in comparison to in Europe (32%) and the United States (5%).^[40]^ Shindo et al^[41]^ report that 13.4% of HCV genotype 1b patients with resistance-proven mutations to PIs were reported in in Japan, while we found 31.1% patients in our study harboring RAVs to PIs.

In addition, we detected that some patients harbor one or more RAVs. Patients who carry combinations of multiple resistance mutations in both or triple the *NS3/4A*, *NS5A*, and *NS5B* genes might increase the possibility of failure in the antiviral treatment with multiple DAA-containing regimens.

This study had certain limitations. The study was held only in our center. The samples of our study were not so large and not all of our cases were successfully amplified.

In conclusion, DAAs RAVs do exist in untreated Chinese patients and the characteristics were different from that in Europe and the United States. R155, A156T, Q80K, and S282T that confer high-level drug resistance were not detected in our study. These results may be associated with the different race and the different HCV genotype epidemiology in our region. A new era of DAAs is now dawning in China; all clinicians should bear in mind that RAVs can pre-exist in HCV1b-infected patient; although the degree of resistance might not be strong, clinicians still need to consider this upon the introduction of DAA-based antiviral therapy. In certain situations, resistance testing might help to select the most optimized treatment option.

## Supplementary Material

Supplemental Digital Content
