# Supplementary material for: Naturally occurring drug resistance associated variants to hepatitis C virus direct-acting antiviral agents in treatment-naive HCV genotype 1b-infected patients in China
Source: Medicine (Baltimore). 2017 May 12;96(19):e6830. doi: 10.1097/MD.0000000000006830 (PMC5428597; doi:10.1097/MD.0000000000006830)
Supplement: Supplemental Digital Content [file medi-96-e6830-s001.docx]

**Supplemental table 1 Primers used for amplifying the core region and NS5B region**

| Gene fragments | Primers | | 5'-3' sequence |
| --- | --- | --- | --- |
| **Core region** | Outer primers | Forward | 5′-ACTGCCTG ATAGGGTGCTTGC-3′ |
|  |  | Reverse | 5′-ATGTACCCCAT GAGGTCGGC-3′ |
|  | Inner primers | Forward | 5'-AGG TCTCGTAGACCGTGCA-3' |
|  |  | Reverse | 5'-CATGTGAG GGTATCGATGAC-3' |
| **NS5B region** | Outer primers | Forward | 5'-CNTAYGGITTCCARTACTCICC-3' |
|  |  | Reverse | 5'-GAG GARCAIGATGTTATIARCTC-3' |
|  | Inner primers | Forward | 5'-TATGAYACCCGCTGYTTTGACTC-3' |
|  |  | Reverse | 5'-GCNGARTAYCTVGTCATAGCCTC-3' |

**Supplemental table 2 Primers used for HCV NS3 amplification by Nested PCR**

| HCV genotype | Primer | 5’- 3’Sequence |
| --- | --- | --- |
| 1b | up1 | GCCGACGGAATGGTCTCCAA |
|  | down1 | CACATCCATCTTGGGCATCGG |
|  | up2 | GGCCTCCTAGGGTGTATAAT |
|  | down2 | GCTTATGACATAATAATTTG |

**Supplemental table 3 Primers used for HCV NS5A amplification by Nested PCR**

| HCV genotype | Primer | 5’­­-3’Sequence |
| --- | --- | --- |
| 1b | up1 | TGCCGGAGAGCGATGCAGCC |
|  | down1 | GAGGTATCATTCAGAGTAGG |
|  | up2 | TCACTGCCATACTCAGCAGC |
|  | down2 | CCTTGCAAGCCCTTGCTGCG |

**Supplemental table 4 Primers used for HCV NS5B amplification by Nested PCR**

| HCV genotype | Primer | 5’­­-3’Sequence |
| --- | --- | --- |
| 1b | Section1up1 | TCATGGTCGACGGTCAGTAG |
|  | Section1down1 | AGTGTAACACCAATAGACAC |
|  | Section1up2 | GGCCGACACGGAAGATGTCG |
|  | Section1down2 | GTGTGGAAAGACCTTCTGGA |
|  | Section2up1  Section2down1  Section2down1  Section2down1 | GGCGTGCGCGTGTGCGAGAA  GACTTGTCCGGTTGGTTCAC  GTACGACGTGGTTAGCAAGC  AGGGCTGCCATATGTGGCAA |
